# Disseminated cryptococcosis with gastrointestinal involvement and false-negative cryptococcal antigen testing due to postzone phenomenon: a case report and review of the literature

**DOI:** 10.1186/s12879-023-08141-y

**Published:** 2023-04-06

**Authors:** Alex N. Zimmet, Grace D. Cullen, Leah Mische, Michael Deftos, Yael Bogler, Nang L. Nguyen, Manoj Ray

**Affiliations:** 1 Division of Infectious Diseases and Geographic Medicine, Stanford University School of Medicine, Stanford, CA USA; 2 Department of Pathology, Santa Clara Valley Medical Center, San Jose, CA USA; 3 Division of Infectious Diseases, Santa Clara Valley Medical Center, San Jose, CA USA; 4 Clinical Microbiology, Santa Clara Valley Medical Center, San Jose, CA USA; 5 Division of Infectious Diseases, Kaiser Permanente Medical Group, Redwood City, CA USA

## Abstract

**Background:**

Cryptococcosis is an increasingly common infection given the growing immunocompromised population worldwide. Cryptococcal antigen (CrAg) testing demonstrates excellent sensitivity and specificity and is the mainstay of diagnosis. However, there may be rare instances in which false-negative CrAg results can delay diagnosis and early treatment, which are critical to ensure positive outcomes.

**Case presentation:**

A 31-year-old man living with HIV/AIDS who was not taking antiretroviral therapy was hospitalized with fever, diarrhea, and headaches. CD4 count on presentation was 71 cells/uL, and HIV viral load was 3,194,949 copies/mL. Serum CrAg testing was initially negative, however CSF CrAg performed several days later was positive at 1:40 and blood and CSF cultures grew *Cryptococcus neoformans*. Colonoscopy revealed mucosal papules throughout the sigmoid colon, and tissue biopsy showed yeast within the lamina propria consistent with GI cryptococcosis. Given the high burden of disease, the original serum CrAg specimen was serially diluted and subsequently found to be positive at 1:2,560, confirming the postzone phenomenon.

**Conclusion:**

Cryptococcosis has a wide array of presentations including intraluminal GI disease, as seen in this patient. While serum CrAg testing displays excellent test characteristics, it is important for clinicians to be aware of the rare instances in which false-negative results may occur in the presence of excess antigen, as in this case.

## Background

*Cryptococcus neoformans* and *Cryptococcus gattii* are basidiomycetous yeast species that cause opportunistic infection in immunocompromised hosts and are a major cause of HIV-related mortality worldwide [1]. As the proportion of the population with immunocompromising conditions grows, it is increasingly important for clinicians across specialties to be familiar with the clinical characteristics, diagnosis, and management of cryptococcosis. The most commonly affected organs are the central nervous system (CNS) and lungs [2] – gastrointestinal (GI) involvement has been rarely reported in humans [3–6].

In all cases, prompt diagnosis and initiation of therapy have a significant impact in avoiding poor outcomes [7]. Cryptococcal antigen (CrAg) testing demonstrates excellent performance characteristics for the diagnosis of cryptococcosis, and serum CrAg measurement has a high predictive value in identifying CNS disease. Measurement of CrAg by lateral flow assay (LFA) is thus a mainstay of diagnosis due to excellent test performance, ease of use, and low cost [8]. Given the centrality of CrAg LFA in the diagnosis and early management of cryptococcosis, clinicians must recognize situations in which false-negative results may occur. There have been reported cases of false-negative LFA in patients with infection due to unencapsulated *Cryptococcus* organisms [9], though this is relatively infrequent. A more common consideration is the postzone effect, in which antigen-antibody binding is impaired due to presence of excessive antigen in the sample leading to a false-negative result. Here we report a case of disseminated cryptococcosis with luminal GI involvement and initial negative serum CrAg testing due to postzone phenomenon. We also review and characterize prior cases of this phenomenon in the literature to promote greater recognition of its potential to confound the diagnostic picture.

## Case presentation

**Fig. 1:**
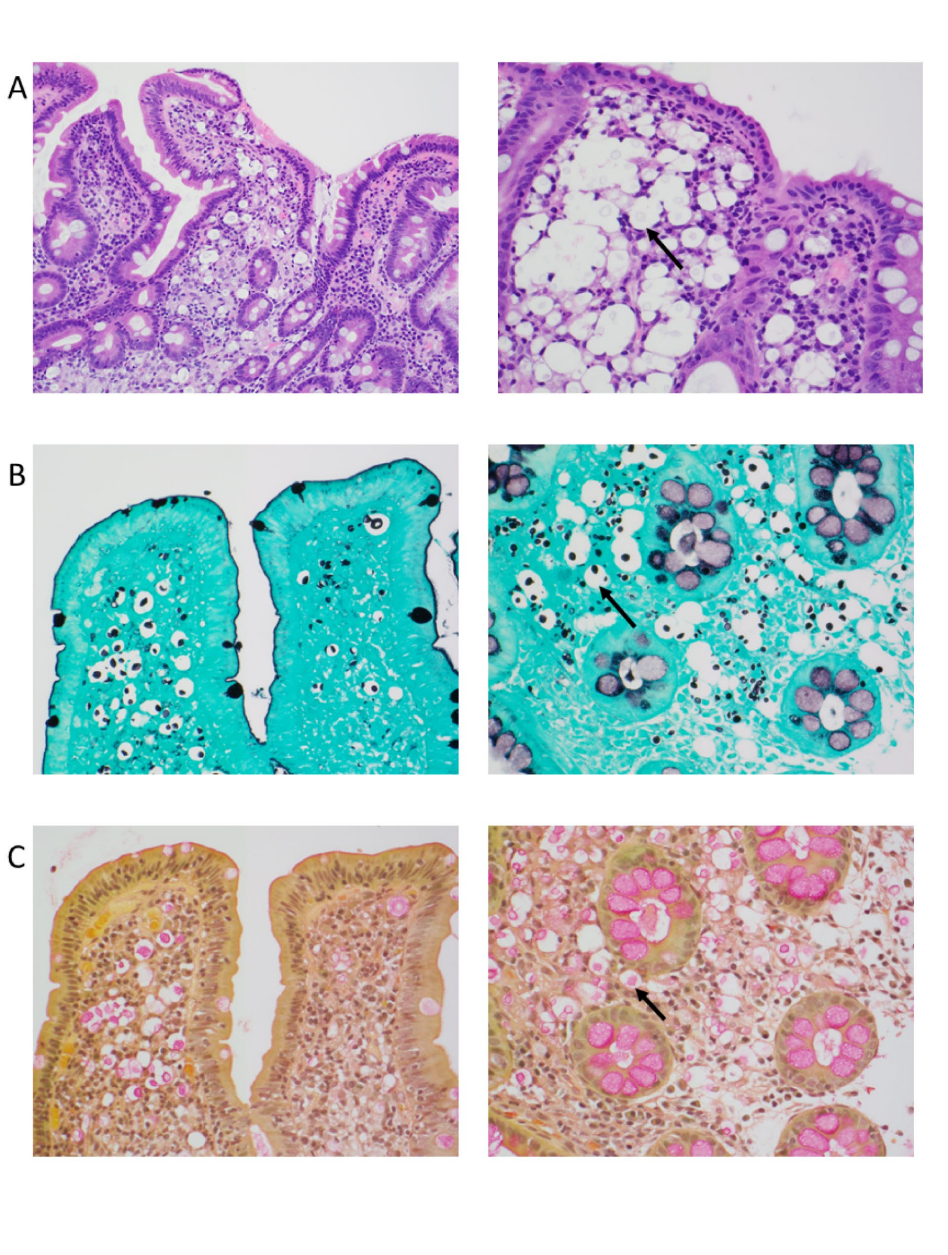
Photomicrographs of mucosal biopsies from the duodenum (left column) and colon (right column) stained with hematoxylin and eosin (panel A), Grocott’s methenamine silver (panel B), and mucicarmine (panel C). Large yeast forms with halos are seen in the lamina propria (arrows), and mucicarmine staining highlights the polysaccharide capsule consistent with encapsulated *Cryptococcus neoformans*

A 31-year-old man living with HIV/AIDS who had not been taking antiretroviral therapy (ART) for several months presented to the Emergency Department with a chief complaint of one month of diarrhea and with recent onset of fevers. He reported at least five liquid, nonbloody bowel movements per day and had measured temperatures as high as 103^o^F. He also complained of headache, but denied focal neurological or respiratory symptoms. He was febrile to 38.2^o^C on presentation, for which he was started on empiric antibiotics. The presenting CD4 T-cell count was 71 cells/uL (6.2%), and the HIV viral load was 3,194,949 copies/mL. A broad diagnostic workup for opportunistic infections was sent; serum CrAg by LFA was reported as negative. Lumbar puncture was initially delayed due to patient hesitancy, however ultimately cerebrospinal fluid (CSF) analysis revealed 4 white blood cells/mcl (normal: 0–5 cells/mcl), protein 23 mg/dl (normal: 10–45 mg/dL), and glucose 45 mg/dl (normal: 40–70 mg/dL). India Ink testing revealed encapsulated budding yeast, and CSF CrAg LFA (IMMY Inc., Norman, OK, USA) was positive at a titer of 1:40. Liposomal amphotericin and oral flucytosine were initiated on day 2 of admission for treatment of cryptococcal meningitis once CSF results were available. Blood and CSF cultures eventually grew *Cryptococcus neoformans.* Esophagogastroduodenoscopy was performed for evaluation of diarrhea and showed pale-appearing but otherwise normal mucosa; random biopsies were taken. Colonoscopy was also performed and revealed multiple small mucosal papules in the sigmoid colon, which were biopsied. Tissue samples from all biopsy specimens showed yeast with clear halos within the lamina propria accompanied by a histiocytic cell reaction; the halos were highlighted on mucicarmine stain, suggesting a mucopolysaccharide capsule and confirming a diagnosis of gastrointestinal cryptococcosis (Fig. 1).

**Fig. 2:**
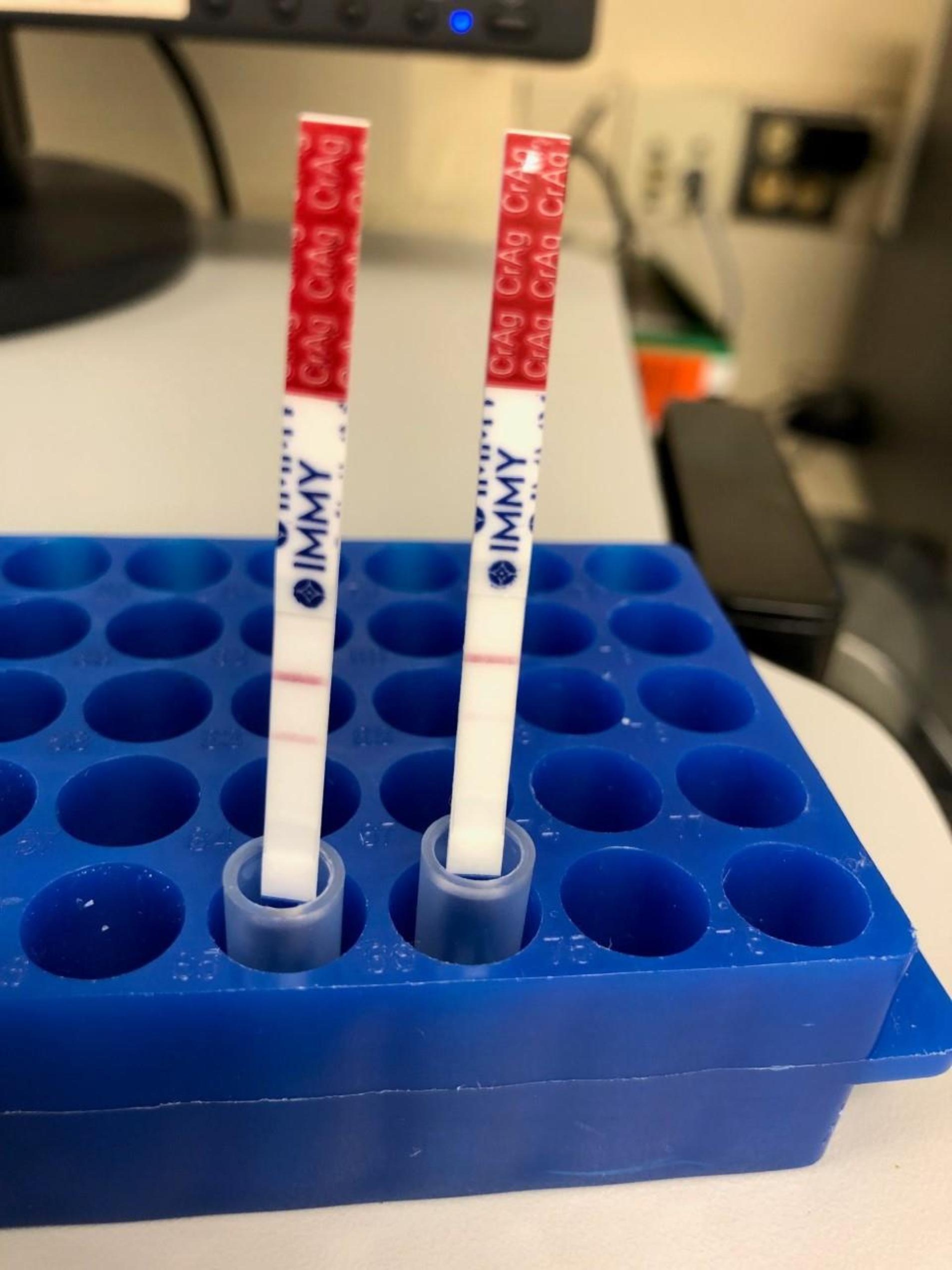
Initial serum CrAg LFA when performed without sample dilution is seen on the right with a very faint positive line, initially read as negative. The left strip demonstrates a more obvious positive result when testing on the same sample was performed at a dilution of 1:160, confirming the postzone phenomenon

The degree of fungal burden and disseminated involvement prompted reconsideration of the initial negative serum CrAg testing. This concern was communicated with the clinical laboratory, and upon re-evaluation of the initial sample a questionable faint positive result was noted. The sample was then diluted 1:160 with a more overt positive result. A final reported titer of 1:2,560 confirmed the postzone phenomenon (Fig. 2).

The patient’s clinical status improved and CSF cultures on antifungal induction therapy. He was subsequently transitioned to fluconazole for consolidation. He initiated ART and established care with the outpatient HIV clinic locally upon discharge. At follow up 3 months later, he was doing well with significant improvement in HIV viral load to 115 copies/mL as well as resolution of fever, headaches, and GI symptoms.

## Discussion and conclusion

We report a case of disseminated cryptococcosis with two especially unusual features – false-negative CrAg testing due to postzone effect and intraluminal GI involvement. *Cryptococcus* species can routinely be isolated from cultures of various clinical specimens, including blood and CSF, when incubated on appropriate media such as Sabourad dextrose agar. However, growth often takes several days, and because CrAg LFA testing typically has excellent performance characteristics and is rapid and cheap, it is by far the favored modality to diagnose cryptococcal infection in clinical laboratories and resource-limited settings [10]. Both clinically-relevant species of *Cryptococcus* possess a polysaccharide capsule external to the cell wall that is a main virulence factor as well as the target of CrAg testing – specifically, the glucuronoxylomannan component of the capsule is specifically detected by most commercially available antigen assays [11].

False-negative CrAg testing is rare; however, false-negative testing due to excess antigen, known as the “postzone” phenomenon, is possible. This is not to be confused with the prozone phenomenon, which describes false-negative testing due to the presence of excess antibody (e.g., when measuring rapid plasma reagin in secondary syphilis) [12]. Together, these phenomena are also known as the “high dose hook effect”. When occurring in cryptococcosis, very high concentrations of CrAg in a sample out-compete the gold-labeled antigen-antibody complex in the test strip for binding of the anti-CrAg monoclonal antibodies in the test line. In the absence of significant binding with the labeled antigen-antibody complex, the test line remains difficult to visualize and is typically read as negative [13]. While the possibility of the hook effect causing false-negative CrAg is listed in the package insert for the most widely used LFA assay [14], in our experience this remains underappreciated by most clinicians and clinical laboratories.

While first reported in 1980 [15], only in recent years has there been increasing recognition in the literature of the postzone phenomenon affecting CrAg testing. We conducted a literature review of all previously reported cases of this phenomenon manifesting with CrAg testing. We identified seven previously published case reports totaling eight patients from various continents worldwide (Table 1). Patients were primarily males (N = 4, 80%) among reports with available demographic data, and 50% of all patients were living with HIV/AIDS. There were two other patients with non-HIV immunocompromising conditions, (one patient undergoing treatment for rheumatoid arthritis, and one patient with nephrotic syndrome), and two patients without documented immune deficits. The postzone phenomenon was seen in clinical samples of both serum (N = 3) and CSF (N = 5). Additionally, while one might presume this false-negative result would only be seen with widespread or multi-organ dissemination given the high fungal burden necessary for it to occur, five (63%) of the reported cases clinically demonstrated isolated organ involvement (CNS or lung) without dissemination [11, 15−17]. In fact, two cases from a single report exhibited the postzone phenomenon in the presence of isolated pulmonary infection with *C. gattii*, which may be more likely to cause disease in immunocompetent hosts than *C. neoformans* [11]. All cases demonstrated markedly high titers when diluted and quantitated.

**Table 1:** Reports of False-Negative Cryptococcal Antigen Lateral Flow Assay Due to Postzone Phenomenon

Reference	Country	Age/Gender	Comorbidities	Site(s) of Disease Involvement	Specimen/Assay	Culture/ Species	Post-Dilution Titer
Stamm and Polt, *JAMA* 1980 [15]	United States	64/M	Rheumatoid arthritis	CNS	CSF/LA	Positive/ *C. neoformans*	1:1,600
Lee et al., *J Clin Microbiol* 2018 [11]	Australia	N/A (2 patients)	None reported	Pulmonary (both patients)	Serum/LFA (both patients)	Positive/ *C. gattii* (both patients)	1:655,360; 1:327,680
Kojima et al., *AIDS* 2018 [16]	Malawi	49/M	HIV/AIDS	CNS	CSF/LFA	Not reported/N/A	Not reported
Yadava and Fazili, *AIDS* 2019 [19]	United States	42/M	HIV/AIDS	Disseminated (CSF, blood, pulmonary)	CSF/LFA	Positive/Not reported	> 1:2,560
Xu et al., *Infect Drug Resist* 2020 [10]	China	79/F	Nephrotic syndrome	Disseminated (CSF, blood)	Serum/LFA	Positive/*C. neoformans*	Not reported
Malik et al., *Ann Clin Lab Sci* 2021 [18]	Malaysia	26/M	HIV/AIDS	Disseminated (CSF, blood)	Serum/LFA, CSF/LFA	Positive/*C. neoformans*	≥ 1:2,560
Tadeo et al., *J Clin Microbiol* 2021 [17]	Uganda	N/A	HIV/AIDS	CSF	CSF/LFA	Positive/Not reported	1:1,310,000

A second important feature of this case is the presence and degree of GI tract involvement. The GI mucosa appeared grossly normal aside from subtle mucosal papules with white center noted in the colon on very close inspection. We posit that these may have been akin to lesions that may be seen on the skin in cryptococcal infection. While intestinal and colonic involvement with *Cryptococcus* has been reported previously, this remains a very rare manifestation of cryptococcosis and is usually asymptomatic or mild disease. The differential diagnosis for causes of diarrhea in persons living with HIV/AIDS is exceptionally wide, and this case further emphasizes how atypical the causes of diarrhea may be in this population. Our case once again highlights the importance of endoscopy with tissue biopsies (even in the presence of normal-appearing mucosa) in the diagnostic workup of this commonly encountered complaint in the immunocompromised host.

Overall, this report and review of the literature underscores that cryptococcosis, one of the most commonly encountered opportunistic infections worldwide, may manifest in numerous organ systems beyond the lungs and CNS. Our case also emphasizes that CrAg testing, while remaining the centerpiece of diagnosis, is not without limitations. In the face of a growing population of immunocompromised individuals cared for across medical subspecialties, clinicians who have a high index of suspicion for cryptococcosis should be aware of the postzone effect as a cause of false-negative CrAg LFA. Serial dilutions may be performed when suspicion is high, as this has been shown to increase sensitivity and specificity to nearly 100% [13]. Lastly, clinicians in general should always question laboratory results that are discordant with a patient’s clinical presentation, be aware of the variety of ways in which false-negative and/or false-positive results may occur for any commonly used testing assay, and readily consult with the serving clinical laboratory to take appropriate and timely action.

## Data Availability

All data generated or analysed during this study are included in this published article.
